# P-653. Child Opportunity Index and RSV Vaccination/Immunization Prior to Hospital Admission

**DOI:** 10.1093/ofid/ofaf695.866

**Published:** 2026-01-11

**Authors:** Lauren Walsh, Jamee Shelley, Tyler Walsh, Jason G Newland

**Affiliations:** Nationwide Children's Hospital, Columbus, OH; Nationwide Children's Hospital, Columbus, OH; Nationwide Children's Hospital, Columbus, OH; Nationwide Children's Hospital, Columbus, OH

## Abstract

**Background:**

Respiratory Syncytial Virus (RSV) causes up to 80,000 hospitalizations in children less than 5 years each year. Two RSV immunoprophylaxis strategies, Nirsevimab for infants and RSV vaccine for pregnant women, are available to protect against serious disease. This study investigates the uptake of these immunoprophylaxis strategies among infants admitted with RSV or non-RSV bronchiolitis. Additionally, we assess the differences of these immunoprophylaxis strategies across Child Opportunity Index (COI).

**Table 1.**
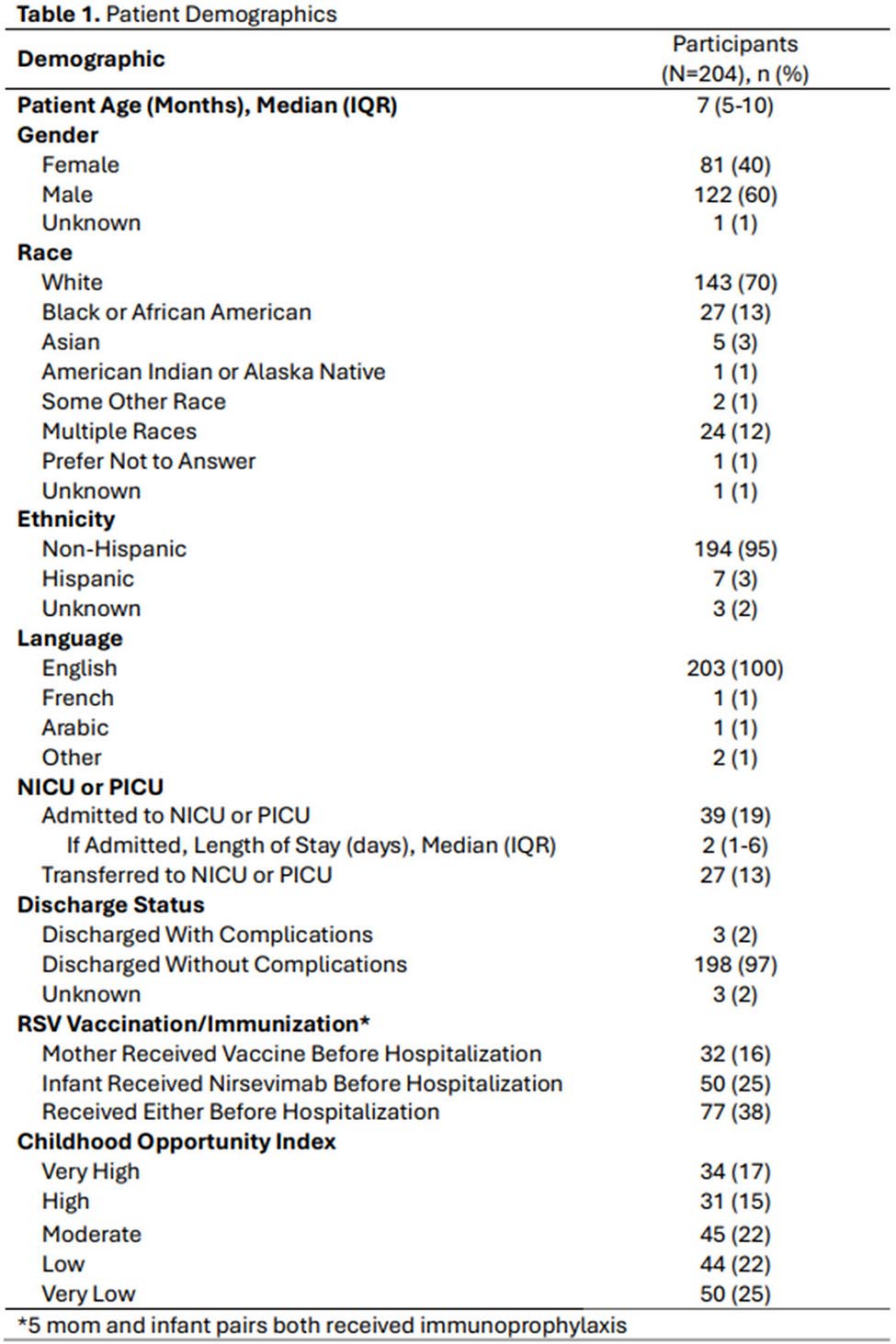
Patient Demographics

**Table 2.**
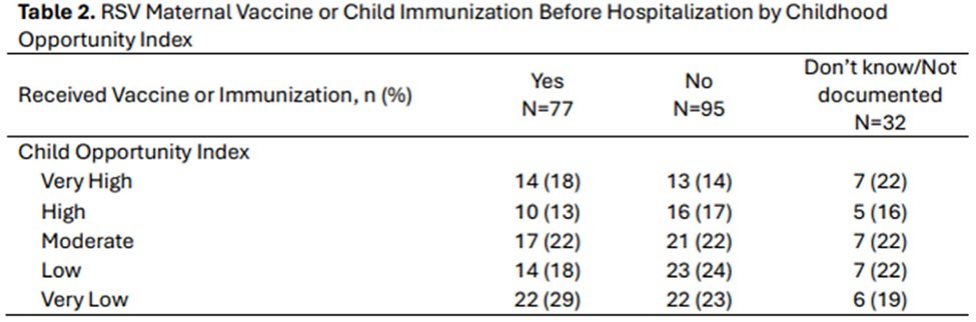
RSV Maternal Vaccine or Child Immunization Before Hospitalization by Childhood Opportunity Index

**Methods:**

Chart reviews were conducted among infants less than 1 year admitted to Nationwide Children’s (NCH) hospital with RSV or non-RSV bronchiolitis between October 2024 – April 2025. Descriptive statistics were used to analyze patient demographics. COIs were matched with patient zip codes to create COI classifications. Maternal vaccination and child immunizations were analyzed across COIs.

**Table 3.**
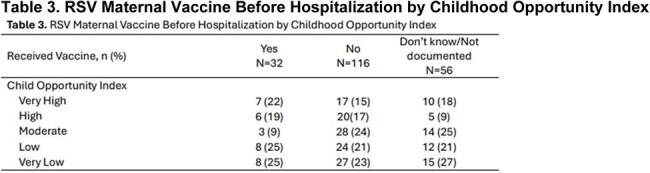
RSV Maternal Vaccine Before Hospitalization by Childhood Opportunity Index

**Table 4.**
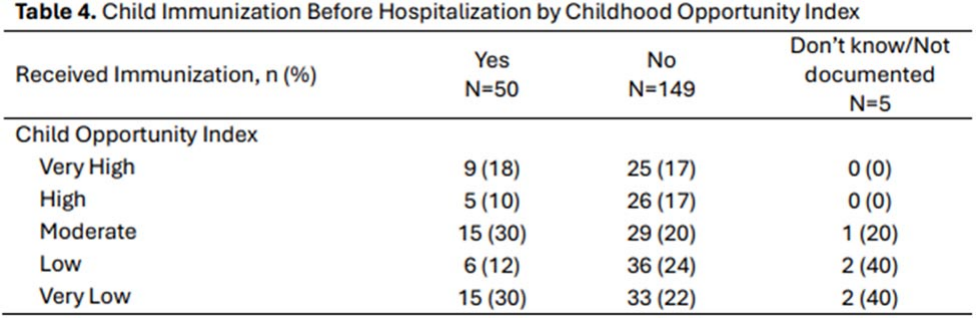
Child Immunization Before Hospitalization by Childhood Opportunity Index

**Results:**

A total of 204 infants were included in the analysis. Most patients were male (60%), white (70%), Non-Hispanic (95%) and a median age of 7 months (IQR 5-10) (Table 1). 16% of moms received immunoprophylaxis before hospitalization, 25% of infants received immunoprophylaxis before hospitalization, and 38% of moms and infants received either of the immunoprophylaxis strategies (Table 1). Five moms and infant pairs both received immunoprophylaxis. 19% of patients were admitted to the NICU or PICU, with a median length of stay in the NICU or PICU of 2 days (Table 1). An additional 13% of the patients were transferred to the NICU or PICU (Table 1). 97% of patients were discharged without complications (Table 1). 47% of patients were from Low or Very Low COI (Table 1) and 47% of patients whose mother received the vaccine or they received the child immunization were from Low or Very Low COI (Table 2).

**Conclusion:**

In this study, more admitted patients were from Low and Very Low COI zip codes and of those who received maternal or child immunoprophylaxis more patients were from Low and Very Low COI zip codes. Further study should be done to understand COI’s role in characterizing RSV hospital admissions and immunoprophylaxis efforts.

**Disclosures:**

Jason G. Newland, MD, MEd, Pfizer: Grant/Research Support

